# Characterisation of full-length cDNA sequences provides insights into the *Eimeria tenella*transcriptome

**DOI:** 10.1186/1471-2164-13-21

**Published:** 2012-01-13

**Authors:** Nadzirah Amiruddin, Xin-Wei Lee, Damer P Blake, Yutaka Suzuki, Yea-Ling Tay, Lik-Sin Lim, Fiona M Tomley, Junichi Watanabe, Chihiro Sugimoto, Kiew-Lian Wan

**Affiliations:** 1 School of Biosciences and Biotechnology, Faculty of Science and Technology, Universiti Kebangsaan Malaysia, 43600 UKM Bangi, Selangor DE, Malaysia; 2 Malaysia Genome Institute, Jalan Bangi, 43000 Kajang, Selangor DE, Malaysia; 3 Institute for Animal Health, Compton, Berkshire, RG20 7NN, UK; 4 Pathology and Infectious Diseases, Royal Veterinary College, University of London, North Mymms, AL9 7TA, UK; 5 Department of Medical Genome Sciences, Graduate School of Frontier Sciences, The University of Tokyo, Kashiwanoha, Kashiwa, Chiba, Japan; 6 Department of Parasitology, Institute of Medical Science, The University of Tokyo, Shirokanedai, Minatoku, Tokyo, Japan; 7 Research Center for Zoonosis Control, Hokkaido University, Hokkaido, Japan

## Abstract

**Background:**

*Eimeria tenella *is an apicomplexan parasite that causes coccidiosis in the domestic fowl. Infection with this parasite is diagnosed frequently in intensively reared poultry and its control is usually accorded a high priority, especially in chickens raised for meat. Prophylactic chemotherapy has been the primary method used for the control of coccidiosis. However, drug efficacy can be compromised by drug-resistant parasites and the lack of new drugs highlights demands for alternative control strategies including vaccination. In the long term, sustainable control of coccidiosis will most likely be achieved through integrated drug and vaccination programmes. Characterisation of the *E. tenella *transcriptome may provide a better understanding of the biology of the parasite and aid in the development of a more effective control for coccidiosis.

**Results:**

More than 15,000 partial sequences were generated from the 5' and 3' ends of clones randomly selected from an *E. tenella *second generation merozoite full-length cDNA library. Clustering of these sequences produced 1,529 unique transcripts (UTs). Based on the transcript assembly and subsequently primer walking, 433 full-length cDNA sequences were successfully generated. These sequences varied in length, ranging from 441 bp to 3,083 bp, with an average size of 1,647 bp. Simple sequence repeat (SSR) analysis identified CAG as the most abundant trinucleotide motif, while codon usage analysis revealed that the ten most infrequently used codons in *E. tenella *are UAU, UGU, GUA, CAU, AUA, CGA, UUA, CUA, CGU and AGU. Subsequent analysis of the *E. tenella *complete coding sequences identified 25 putative secretory and 60 putative surface proteins, all of which are now rational candidates for development as recombinant vaccines or drug targets in the effort to control avian coccidiosis.

**Conclusions:**

This paper describes the generation and characterisation of full-length cDNA sequences from *E. tenella *second generation merozoites and provides new insights into the *E. tenella *transcriptome. The data generated will be useful for the development and validation of diagnostic and control strategies for coccidiosis and will be of value in annotation of the *E. tenella *genome sequence.

## Background

Coccidiosis is an economically important intestinal disease of poultry caused by parasitic *Eimeria *species. The annual cost of coccidiosis to the poultry industry worldwide has been estimated to exceed £2 billion [1]. Control of this disease in intensively reared poultry is accomplished principally by prophylactic chemotherapy with specific anticoccidial drugs, although drug-resistance is a serious problem that has to be constantly managed. No new drugs have been introduced in recent years and alternative methods of control are now required. Vaccination using live vaccines is a viable option, though it is hampered by the complexity and production constraints of live parasites. Thus, new approaches for control continue to be sought.

*Eimeria tenella *is widely considered to be the most economically relevant and well known of the seven *Eimeria *species that cause coccidiosis in chickens [2]. The second generation merozoite of *Eimeria *is an interesting target for transcriptomic studies as it is the progeny derived from the most pathogenic endogenous stage of the *E. tenella *life cycle [3] and may contribute to the stimulation of the protective immune response in the host for at least some *Eimeria *species [4]. In addition, it is among the most readily isolated stages of the life cycle [5]. Detailed study of the merozoite stage will support the identification of proteins important to key biological processes in the parasite including host invasion, replication, pathogenicity and the stimulation of host immunity.

The availability of segments of sequences from randomly selected cDNA clones, known as expressed sequence tags (ESTs), has provided valuable resources for the identification and study of genes in *E. tenella *[6-8]. Sequencing of full-length cDNAs provides additional advantages including data derived from a single clone rather than an assembly of multiple ESTs, which can generate ambiguous contigs, and complete transcripts, which include open reading frames (ORFs) and untranslated regions (UTRs). Thus, a large collection of full-length cDNA sequences provides a set of protein coding sequences that facilitate the prediction of gene identity and function by comparison with other known protein coding genes [9].

In this study, partial sequences were generated from the 5' and 3' ends of randomly selected clones of an *E. tenella *second generation merozoite full-length cDNA library. These partial sequences were pre-processed and subsequent sequence clustering and primer walking generated full-length cDNA sequences. Characterisation of these full-length cDNA sequences included determination and analysis of ORFs and UTRs, Kozak sequence consensus, simple sequence repeats (SSRs) and codon usage. Analysis of the full-length cDNA sequences generated also identified candidate secretory and membrane proteins that may prove relevant in developing disease control strategies against avian coccidiosis.

## Results and discussion

### Generation of full-length cDNA sequences

A total of 9,024 clones were randomly selected for plasmid extraction and subsequent single-pass sequencing from the 5' and 3' ends. After eliminating low quality and vector contaminated sequences, 8,433 and 6,981 good quality sequences were obtained from the 5' and 3' ends respectively [dbEST: JK017416-JK032828, JK032875]. These partial sequences were clustered and resulted in the identification of 1,529 unique transcripts (UTs). Using the clustered sequences 81 full-length cDNA sequences were generated by aligning overlapping 5' and 3' end partial sequences. In addition, clones representing 586 consensus sequences with both 5' and 3' end partial sequences were randomly selected and subjected to complete sequencing by single-pass primer walking, generating a further 363 full-length cDNA sequences. Primary sequence analysis revealed the absence of in-frame start or stop codons in one and 10 clones respectively. Such sequences might represent non-coding RNAs, although they could also have been derived from contaminants or cloning artefacts and have been excluded from our subsequent analyses. Thus, a total of 433 full-length cDNA sequences were generated and analysed in this study [GenBank: JN987230-JN987662].

### Functional annotation

**Figure 1:**
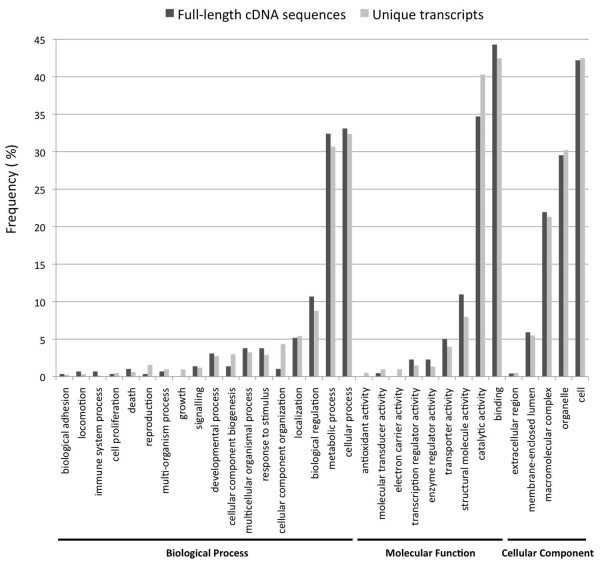
**Distribution of GO terms of unique and full-length transcripts of *Eimeria tenella***.

BLASTX similarity search of the 1,529 UTs against the GenBank non-redundant database revealed that 54.2% (829/1,529) of the transcripts had significant matches (E-value < 1e-6) to publicly available gene sequences, with most of these (71.4%) matches to gene sequences from apicomplexan parasites [Additional file 1]. A total of 2,053 gene ontology (GO) terms, distributed within the categories Biological Process, Molecular Function and Cellular Component, were assigned to the 1,529 UTs (831, 603 and 619 respectively) [Figure 1]. The most highly represented subcategories within Biological Process were cellular and metabolic processes, accounting for 32.4% (269/831) and 30.7% (255/831) of the transcripts respectively, in line with previous proteomic characterisation of the second generation merozoite [10]. Binding (42.5%; 256/603) and catalytic activity (40.3%; 243/603) were the most highly represented subcategories within Molecular Function. Combined, these results are consistent with the biological role of the second generation merozoite, a life cycle stage characterised by metabolically active processes including motility within the caeca, host cell attachment and cellular invasion. The Cellular Component category was dominated by the cell (42.5%; 263/619) and organelle (30.2%; 187/619) subcategories, consistent with the relatively high abundance of cell cycle-associated proteins reported in the second generation merozoite proteome compared to the sporozoite [10]. GO annotation of the *E. tenella *full-length cDNA sequences revealed similar functional patterns [Figure 1; Additional file 2].

### cDNA, ORF and UTR length distribution

**Figure 2:**
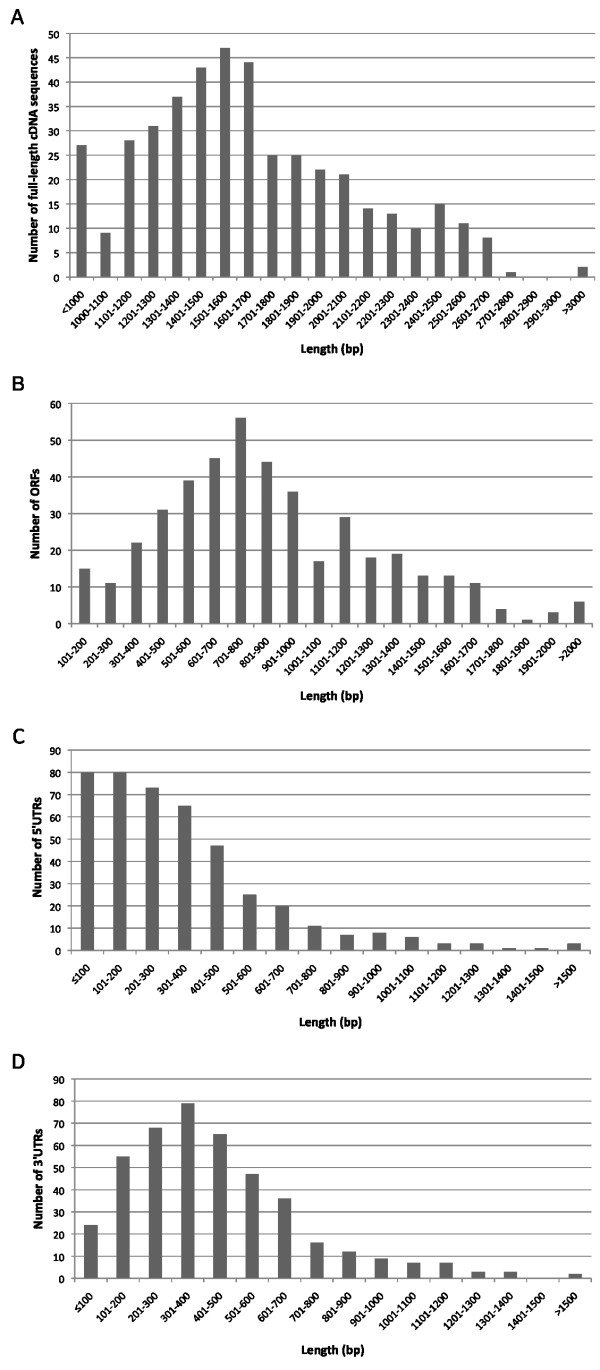
**Length distribution of (A) full-length cDNA sequences, and (B) ORF, (C) 5'UTR and (D) 3'UTR of the full-length cDNA sequences of *Eimeria tenella***.

Analysis of the 433 *E. tenella *full-length cDNA sequences revealed an average size of 1,647 bp [Figure 2]. Most of the sequences were in the length range of 1,401 bp to 2,100 bp, while 24.2% (105/433) of the sequences were within 1,000 bp to 1,400 bp and 16.6% (72/433) were within 2,101 bp to 3,000 bp. The analysis also showed that 27 sequences generated were less than 1,000 bp in length and two sequences were more than 3,000 bp in length. Our analysis of the previously reported 732 full-length cDNA sequences from *Toxoplasma gondii *[11] and 644 full-length cDNA sequences from *Cryptosporidium parvum *[12] revealed average sizes of 1,539 bp and 1,399 bp respectively. Comparison of the three data sets revealed longer average full-length cDNA sequences within the *E. tenella *transcriptome. While the difference was not statistically significant, variation in transcriptome-wide SSR prevalence between the three data sets is clearly a contributory factor (described in detail below).

The sequences were also analysed to predict ORF and UTR components following start and stop codon identification [Additional file 3]. Length distribution analysis of the 433 predicted ORFs showed that the majority were between 501 to 900 bp in length, with the average size being 867 bp [Figure 2]. Approximately 18.2% (79/433) of the ORFs were less than 500 bp, while only 1.4% (6/433) of the ORFs were more than 2,000 bp. The length distribution of the 5'UTRs showed that few exceeded 500 bp (20.3%; 88/433), with the average size of the 5'UTR being 342 bp. The length distribution of the 3'UTRs showed that 9.9% (43/433) were more than 800 bp long, with the average size of the 3'UTR being 438 bp.

Although the 5'UTR does not contribute directly to the encoded protein, the characterisation of 5'UTR features is important as this region is believed to be involved in the control of translation and transcription processes that subsequently reflect gene expression [13,14]. Thus, data generated on these regions may reveal control elements and regulatory mechanisms of gene expression patterns in the parasite. In a previous study of apicomplexan full-length cDNA sequences, Wakaguri et al [11] reported that the average size of 5'UTRs was consistent amongst *Plasmodium *species, namely *P. falciparum *(303 bp), *P. vivax *(304 bp), *P. yoelii *(345 bp) and *P. berghei *(299 bp), but varied between genera with *C. parvum *and *T. gondii *presenting average 5'UTR lengths of 137 bp and 288 bp respectively. The average 5'UTR length was shortest for *C. parvum*, which may reflect the fact that both the genome size and the average gene size are the smallest in this species. This comparison has revealed longer 5'UTRs in the *E. tenella *genome than reported for most other apicomplexan parasites. While the significance of this finding is not yet clear the detection of numerous SSRs may once again be a contributory factor.

### Genomic cDNA transcript mapping-*E. tenella *chromosome 1

*Eimeria tenella *is the first of the *Eimeria *species parasites to have been subjected to genome sequencing, although the draft assembly remains fragmented [15]. In order to demonstrate the utility of the data generated here for gene prediction the 1,529 UTs and 433 full-length cDNA sequences were mapped onto the first sequenced *E. tenella *chromosome (chromosome 1), representing ~1.8% of the genome [16]. Based on an overlap of at least 70% of the original transcript length, a total of 13 UTs were successfully mapped-seven to genes in the feature-poor 'P'-regions and the remaining six to genes in the feature-rich 'R'-regions of the chromosome [Additional file 4; Additional file 5]. Further analysis revealed that mapping of the UT sequences identified and resolved several inconsistencies with the previously predicted coding regions, indicating the usefulness of the transcript sequences in improving gene structure prediction on the *E. tenella *genome sequence. Two full-length cDNA transcripts were mapped to *E. tenella *chromosome 1 [Additional file 6] where the alignment of ln23_Etm023C06 showed consistency with the previously characterised 15-exon structure of the glucose-6-phosphate isomerase gene [16,17].

### SSR motif analysis

SSRs can be found in the genome of both prokaryotic and eukaryotic organisms [18,19]. These repeats represent a rich source of hypervariable markers due to the constant allelic changes of array length caused by their high mutation rate [20,21]. As a result, they have been widely used in the fields of linkage mapping [22,23], population genetics [24] and phylogenetic or comparative genomic research [25,26]. In addition, SSRs are believed to be important in genome evolution, stimulating the development of genetic variability [27] and influencing gene expression [28,29].

A notable feature of *E. tenella *chromosome 1 was the abundance of SSRs, not only in the introns and intergenic regions, but also in the predicted coding regions [16]. In order to further characterise the type and location of these repeated motifs in *E. tenella *genes, SSR motif analysis was carried out on the full-length cDNA sequences generated. Results showed that the SSRs present were composed of various types of mono-, di-, tri-, tetra-, hexa-, hepta-, nona- and decanucleotides [Table 1; Additional file 7]. The location of the SSRs were subsequently categorised to three different locations, i.e. 5'UTR, ORF and 3'UTR. Based on the distribution of the SSRs trinucleotide motifs were found to be the most abundant (88.4%; 455/515), with mono- and tetranucleotide motifs also found to be common. The trinucleotide CAG was the most abundant motif and constituted 71.4% (325/455) of the entire trinucleotide motifs identified, while the most abundant tetranucleotide motif was AGCT, which comprised of 71.4% (15/21) of all tetranucleotide motifs. A total of eight hexanucleotide, two heptanucleotide, two nonanucleotide and one decanucleotide motifs were also identified in the *E. tenella *full-length cDNA sequences. The abundance of CAG trinucleotide motifs within the *E. tenella *full-length cDNA sequences was consistent with the published findings from *E. tenella *chromosome 1 [16]. Comparison with publicly available full-length cDNA sequences from *C. parvum *[12] and *T. gondii *[11] revealed the dominance of mononucleotide repeats for the former, but dinucleotide repeats for the latter [Additional file 8]. Surprisingly, *T. gondii *full-length cDNA sequences were found to contain only mono-, di- and trinucleotide repeats, while penta-, hepta-, octa-, nona- and decanucleotide motifs were also found to be absent in *C. parvum*. As expected, the highest SSR content was observed in *E. tenella*.

**Table 1:** SSR motif distribution in full-length cDNA sequences of *Eimeria tenella*

SSR type	5'UTR	ORF	3'UTR	Total
Mononucleotide	8	4	6	18
Dinucleotide	-	1	7	8
Trinucleotide	133	188	134	455
Tetranucleotide	-	-	21	21
Hexanucleotide	1	7	-	8
Heptanucleotide	2	-	-	2
Nonanucleotide	-	1	1	2
Decanucleotide	-	-	1	1

Total	144	201	170	515

### Codon usage analysis

Codon usage often varies between organisms and may reflect the cellular location of gene products [30] and aid in coding region determination [31]. Codon usage in both eukaryotes and prokaryotes is known to be affected by directional mutation of nucleotides present in the genome [32] and may be influenced by the composition of a genome's transfer RNA (tRNA) portfolio. Thus, genomic evolution frequently demonstrates genome-specific over- or under-representation of some dinucleotides, and dinucleotide frequency is believed to influence codon usage [33,34]. Dinucleotides that are under-represented in coding regions thus appear as codons that are present at low frequency. In this study, the ORFs identified were subjected to codon usage analysis using CodonW and a codon usage table for full-length *E. tenella *ORFs was subsequently generated consisting of 125,231 codons [Table 2]. A previous study of codon usage by parasites including *Babesia bovis*, *Theileria parva*, *T. gondii *and *E. tenella *showed that codons CGA, CGG and UGU are infrequently used by all of these organisms [35]. For *E. tenella*, it was revealed that based on the frequency of usage of less than 10 per 1000 codons, 17 codons (GUA, AGA, AGU, AUA, ACG, UGU, UAU, UUA, UUU, UCG, UCA, CGG, CGA, CGU, CAU, CAC and CUA) are infrequently used. Six of these codons contain either the UA or AU dinucleotide, while another four contain the CG dinucleotide. In this study, analysis of the *E. tenella *ORF sequences identified ten codons (UAU, UGU, GUA, CAU, AUA, CGA, UUA, CUA, CGU and AGU) that are infrequently used based on the same criterion. Comparison between these two studies revealed that all ten of the codons identified in this study are the same as those identified by Ellis et al [35]. Furthermore, six of them also contained either the UA or AU dinucleotide while two of them contained the CG dinucleotide, supporting the finding of the previous study that codons with low usage frequency contain under-represented UA, AU or CG dinucleotides. Five codons were over-represented (GCA, CAG, AAG, GAA and GAG), although for GCA and CAG this may have been skewed by the SSR distribution.

**Table 2:** Codon usage in full-length coding sequences of *Eimeria tenella*

AA	Codon	Frequency	Codon Usage Value*	AA	Codon	Frequency	Codon Usage Value*
Phe	UUU	2044	16.32	Tyr	*UAU*	*968*	*7.73*
	UUC	2545	20.32		UAC	1726	13.78
Leu	*UUA*	*695*	*5.55*	TER	UAA	121	0.97
	UUG	2262	18.06		UAG	114	0.91
	CUU	2067	16.51	His	*CAU*	*926*	*7.39*
	CUC	2224	17.76		CAC	1715	13.69
	*CUA*	*986*	*7.87*	Gln	CAA	2156	17.22
	CUG	3595	28.71		**CAG**	**4176**	**33.35**
Ile	AUU	1949	15.56	Asn	AAU	1480	11.82
	AUC	1592	12.71		AAC	2301	18.37
	*AUA*	*1049*	*8.38*	Lys	AAA	2786	22.25
Met	AUG	2553	20.39		**AAG**	**4160**	**33.22**
Val	GUU	2001	15.98	Asp	GAU	2237	17.86
	GUC	1886	15.06		GAC	3588	28.65
	*GUA*	*933*	*7.45*	Glu	**GAA**	**4165**	**33.26**
	GUG	2951	23.56		**GAG**	**4561**	**36.42**
Ser	UCU	1806	14.42	Cys	*UGU*	*681*	*5.44*
	UCC	1413	11.28		UGC	1703	13.60
	UCA	1297	10.36	TER	UGA	198	1.58
	UCG	1285	10.26	Trp	UGG	1299	10.37
Pro	CCU	1777	14.19	Arg	*CGU*	*938*	*7.49*
	CCC	1970	15.73		CGC	1952	15.59
	CCA	1719	13.73		*CGA*	*926*	*7.39*
	CCG	1579	12.61		CGG	1331	10.63
Thr	ACU	1716	13.70	Ser	*AGU*	*1002*	*8.00*
	ACC	1263	10.09		AGC	2825	22.56
	ACA	1734	13.85	Arg	AGA	1420	11.34
	ACG	1588	12.68		AGG	1393	11.12
Ala	GCU	3388	27.05	Gly	GGU	1252	10.00
	GCC	2905	23.20		GGC	3142	25.09
	**GCA**	**4159**	**33.21**		GGA	2125	16.97
	GCG	2926	23.36		GGG	2007	16.03

*Codon usage values given are the frequency per 1000 codons.

Under-represented codons (frequency of usage less than 10 per 1000 codons) are in italics while over-represented codons (frequency of usage more than 30 per 1000 codons) are in bold.

### Determination of consensus sequence of translational initiation sites from full-length cDNA sequences of *E. tenella*

Start codons derived from the 433 full-length cDNA sequences were aligned to observe the consensus sequence of translational initiation sites (the Kozak sequence) in *E. tenella*. A clear consensus sequence was identified with A dominating at positions -3, -2 and -1, while G dominated at position +4. However, at position -4, two bases ie. C and G were found to co-dominate. Thus, the Kozak sequence (G/C)AAAATGG can be assigned for *E. tenella *genes [Additional file 9]. In a previous study [36], a total of 26 *T. gondii *gene sequences from position -20 relative to the ATG start codon up to position +4 were compared. A consensus sequence was apparent with A dominating at positions -3, -2 and -1, plus C at position -4 and G at position +4. Thus, the Kozak sequence CAAAATGG was assigned for *T. gondii*. Comparison of the Kozak sequences for *E. tenella *and *T. gondii *shows a high similarity where A at positions -3, -2 and -1, and G at position +4 for both organisms are similar. However, the Kozak sequence for both of these organisms appears to differ from that of the higher eukaryotes [37] [Table 3].

**Table 3:** Comparison of Kozak motif consensus sequences between *Eimeria tenella*, *Toxoplasma gondii *and higher eukaryotic organisms

Position	-4	-3	-2	-1	ATG	+4	Source
*E. tenella*	C/G	A	A	A	ATG	G	In this study
*T. gondii*	C	A	A	A	ATG	G	[36]
Eukaryote	C	A/G	C	C	ATG	G	[37]

### Secretory and membrane protein prediction

Parasite secreted proteins commonly interact with host cells at the molecular level and are exposed to the host immune system. Parasite growth and invasion processes may be prevented once an essential secretory protein is inhibited. Therefore, many secretory proteins can be considered to be vaccine candidates or potential drug targets [38-41]. Prediction analysis using SignalP suggested that 19.6% (85/433) of the peptide sequences contain a signal peptide. Out of these 85 peptide sequences, 60 were predicted to contain one or more transmembrane domains and/or a GPI-anchor, leaving 25 as predicted unbound secretory proteins. Similarity searches based upon homology showed that a large proportion of these predicted secretory proteins could not be assigned a putative function as 24.0% (6/25) had matches with hypothetical proteins or proteins with unknown function, while 56.0% (14/25) had no significant similarity to any publicly available protein sequence [Additional file 10]. Intriguingly, although most of the putative secretory proteins identified were apparent homologues of apicomplexan genes no recognised apical organellar proteins were found.

Many apicomplexan surface proteins have been shown to play an important role in the pathogenicity of these parasites and a number of them are potential vaccine candidates or drug targets. Proteins that are attached via a GPI-anchor to the surface of protozoan parasites can induce a variety of host immunological responses [42,43]. In this study, membrane proteins were predicted by identifying the presence of signal peptides, transmembrane domains and GPI-anchors. The prediction of transmembrane helices carried out using TMHMM revealed a total of 92 peptide sequences likely to contain at least a single transmembrane domain. GPI-anchor prediction analysis carried out using GPI-SOM, which detects both the N-terminal signal peptide and C-terminal GPI-anchor signal, suggested a total of 26 peptides with a GPI-anchor. Protein sequences that contain a signal peptide and a transmembrane domain or a GPI-anchor were predicted to be membrane proteins. Based on these criteria, 60 membrane proteins were predicted in this study. Database similarity searches showed that putative functions could not be assigned to most of the predicted membrane proteins as 5.0% (3/60) were most similar to hypothetical proteins, while 48.3% (29/60) had no significant similarity with sequences in the GenBank database [Additional file 11]. In total 31.7% (19/60) of the predicted membrane proteins had matches with *E. tenella *surface antigens (EtSAGs). Two proteins had a perfect match with members from the previously described A family (i.e. EtSAG4 and EtSAG6) [44]. Interestingly, seven other predicted surface proteins showed between 45.8% and 95.5% similarity to the entire coding region length of the EtSAGs. Using multiple sequence alignment these sequences can be divided into two groups, representing the A and B families [Additional file 12]. The alignments show the presence of the six conserved cysteine residues in both families. Family A revealed a mosaic pattern with conserved and variable regions distributed throughout the alignment while family B exhibited a more biased pattern with variation predominantly in the N-terminal half of the alignment, consistent with the analysis described by Tabares et al. [44]. This analysis strongly suggests that the surface antigens discovered in this study represent new members of the EtSAG families. Both of the previously annotated EtSAGs identified in this study had been reported to be expressed in second generation merozoites [44]. Using GO many of the other putative membrane proteins were classified as involved in cellular and metabolic processes; for example identification of a putative longevity-assurance (LAG1) domain-containing protein. As described elsewhere such molecules can present opportunities to disrupt parasite infection and thus have the potential to become good targets for novel intervention strategies [45].

## Conclusions

In this study, we generated and analysed 433 full-length cDNA sequences with complete coding regions derived from the *E. tenella *second generation merozoite transcriptome. These sequences provide access to a relatively large resource of nucleotide and amino acids sequences for *E. tenella *that will support a better understanding of the transcriptome of this economically relevant parasite. Moreover, in combination with other genomic resources including whole genome sequences and genome maps [46], these full-length cDNA sequences will offer new insights into the structure, composition and function of the *E. tenella *genome. We have also identified panels of 25 and 60 predicted secretory and membrane proteins, with potential for development as novel diagnostic and/or control strategies for *E. tenella *via molecular techniques.

## Methods

### Parasite passage and purification

The reference *E. tenella *Houghton strain was used throughout this study [2]. The parasite was routinely propagated as described elsewhere [5] using specific pathogen free Light Sussex chickens produced and maintained at the Institute for Animal Health. Second generation merozoites were purified following the method of Prof. N. Smith as described elsewhere using several serial five minute incubation steps, each in fresh incubation medium [5]. Only incubation medium washes lacking microscopically detectable red blood cells were processed for RNA extraction to limit host cell contamination.

### Full-length cDNA library construction

RNA was extracted from *E. tenella *second generation merozoites using the TRIzol reagent as described by the manufacturer (Invitrogen, USA) and used in the construction of a full-length cDNA library by the oligo-capping method [47]. In brief, RNAs were sequentially treated with bacterial alkaline phosphatase (BAP) and tobacco acid pyprophosphatase (TAP). The BAP-TAP treated RNAs were then ligated with 5' oligo-cap linker using RNA ligase. First strand cDNAs were synthesised with the oligo-capped mRNA as a template, followed by PCR using the oligo-cap linker sequence and oligo-dT-adapter as primers. The full-length cDNAs produced were then cloned into the pME18S-FL3 plasmid vector and subsequently transformed into *Escherichia coli *ElectroMAX DH10B cells (Invitrogen, USA).

### Plasmid extraction and cDNA sequencing

Colonies were picked randomly and inoculated into individual wells of 96-deep well plates containing LB media, and subsequently grown overnight. Plasmid DNAs were extracted using the Montage™ Plasmid MiniPrep_96 _Kit (Milipore, USA) according to the manufacturer's instructions. The cDNA inserts were sequenced once from the 5' and 3' ends using the forward (5' GGA TGT TGC CTT TAC TTC TA 3') and reverse (5' TGT GGG AGG TTT TTT CTC TA 3') primers respectively, and the Big Dye Terminator v3.1 Cycle Sequencing Kit (Applied Biosystem Inc., USA) on an ABI PRISM 3730×l DNA Analyzer (Applied Biosystem Inc., USA).

### Generation of full-length cDNA sequences

The generated 5' and 3' end sequences were pre-processed using a Phred [48,49] cut-off quality value of 20. The sequences were subsequently screened against the GenBank non-redundant nucleotide database, and specifically against chicken genome sequences. No sequences with more than 90% similarity to a known chicken genome sequence were identified. Clustering was then carried out using StackPACK version 2.2 [50,51]. Consensus sequences with overlapping 5' and 3' end sequences were identified as representing full-length cDNA sequences, while those containing both the 5' and 3' end sequences that did not overlap were selected for single-pass primer walking to generate full-length cDNA sequences. Internal primers for primer walking were designed using Primer3 [52]. The sequence reads generated were manually assembled to produce a consensus sequence with a coverage of at least one strand.

### Functional annotation and mapping of transcript sequences

The consensus and full-length cDNA sequences were compared against the GenBank non-redundant database using BLASTX [53], and the assignment of GO terms was carried out using the BLAST2GO pipeline [54]. Mapping of UTs and the full-length cDNA sequences to *E. tenella *chromosome 1 [16] was carried out separately using ssahaEST [55] with the following parameters: kmer = 10, seeds = 3, skip = 10, cutp = 80, score = 40, depth = 50, memory = 40, array = 0, edge = 200, identity = 95. Each transcript aligned to the chromosome 1 sequence was required to include at least 70% of the original transcript sequence and mapped in a single contiguous sequence without non-intron/exon gaps. Single-exon alignments were required to include at least 50 bp, while in multi-exon alignments, each aligned exon was required to be longer than 10 bp, with introns between 5 bp to 5000 bp. The transcript mapping results were inspected manually using the Artemis genome browser [56].

### Characterisation of ORFs and UTRs

The coding region in each full-length cDNA sequence was individually predicted using ORF Finder [57]. Whenever possible, BLAST matches were used to confirm the reading frame, and in-frame start and stop codon positions. The determined ORFs and UTRs were analysed with MISA [58] to identify and localise SSRs. The coding regions were also submitted to CodonW [59] to generate a codon usage table. Kozak sequence consensus analysis was carried out by generating sequence logos using WebLogo [60].

### Secretory and membrane protein prediction

Secretory and membrane proteins were predicted using SignalP 4.0 [61] and TMHMM 2.0 [62]. GPI-anchored proteins were predicted using GPI-SOM [63], which predicts both the N-terminal signal peptide and C-terminal GPI-anchor signal. Protein localisation analysis using WoLF PSORT [64] and BLAST matches were used to support each prediction.

The bioinformatic tools used in this study are summarised in Additional file 13.

## Authors' contributions

DPB, FMT, JW and K-LW conceptualised the research plan. YS constructed the full-length cDNA library. NA generated the partial and full-length cDNA sequences, and together with X-WL, Y-LT and L-SL analysed the data. DPB, YS, JW, CS and K-LW participated in data collection monitoring and data interpretation. NA and X-WL drafted the manuscript. DPB, FMT and K-LW critically revised the manuscript. K-LW supervised and coordinated the study. All authors read and approved the final manuscript.

## Supplementary Material

Additional file 1 **BLASTX results of *Eimeria tenella *unique transcripts**. List of *Eimeria tenella *unique transcripts with significant matches to sequences in the non-redundant GenBank database together with the corresponding E value, accession number, putative identity and organism. Click here for file

Additional file 2 **BLASTX results of *Eimeria tenella *full-length cDNA sequences**. List of *Eimeria tenella *full-length cDNA sequences with significant matches to sequences in the non-redundant GenBank database together with the corresponding E value, accession number, putative identity and organism. Click here for file

Additional file 3 **Details of the ORFs, 5'UTRs and 3'UTRs of *Eimeria tenella *full-length cDNA sequences**. List of *Eimeria tenella *full-length cDNA sequences together with their corresponding details including the nucleotide sequence and length of the full-length cDNAs and their ORF, translated ORF, 5'UTR and 3'UTR sequences and lengths. Click here for file

Additional file 4 **Mapping of *Eimeria tenella *transcripts to chromosome 1 sequence**. Number of *Eimeria tenella *unique and full-length transcripts mapped to the predicted coding, P- and R-regions of chromosome 1. Click here for file

Additional file 5 **Position of *Eimeria tenella *transcripts on chromosome 1 sequence**. Graphical representation of the positions of *Eimeria tenella *(A) unique and (B) full-length transcripts that mapped to chromosome 1. Segmentation of the chromosome is shown in cyan (P-region) and blue (R-region). Positions of transcripts are represented by red vertical lines. Click here for file

Additional file 6 **Alignment of *Eimeria tenella *full-length transcripts to chromosome 1**. Graphical representation of the alignment of full-length transcripts (A) ln214_Etm109D12 and (B) ln23_Etm023C06 to the respective predicted genes on chromosome 1. Mapped full-length transcripts are shown in red while predicted genes are shown in cyan. Click here for file

Additional file 7 **Details of SSR motif distribution in full-length cDNA sequences of *Eimeria tenella***. List of SSR motifs identified in *Eimeria tenella *full-length cDNA sequences together with their repeat number, copy number and total length within the 3'UTR, ORF and 5'UTR. Click here for file

Additional file 8 **Comparison of SSR motifs in *Eimeria tenella*, *Toxoplasma gondii *and *Cryptosporidium parvum *full-length cDNA sequences**. List of SSR motifs identified in *Eimeria tenella*, *Toxoplasma gondii *and *Cryptosporidium parvum *full-length cDNA sequences together with their repeat number, copy number, total length and the percentage of their total length over the total length of the respective full-length cDNA sequences. Click here for file

Additional file 9 **Kozak motif profile from full-length cDNA sequences of *Eimeria tenella***. Graphical representation of the consensus sequence of translational initiation sites (the Kozak sequence) based on the alignment of start codons derived from *Eimeria tenella *full-length cDNA sequences. Click here for file

Additional file 10 **Secretory proteins predicted from the full-length cDNA sequences of *Eimeria tenella***. List of *Eimeria tenella *full-length cDNA sequences predicted to code for secretory proteins together with the corresponding evidences based on the results of SignalP, TMHMM, GPI SOM and WoLF PSORT analyses, and details of functional annotation based on BLASTX similarity search. Click here for file

Additional file 11 **Membrane proteins predicted from the full-length cDNA sequences of *Eimeria tenella***. List of *Eimeria tenella *full-length cDNA sequences predicted to code for membrane proteins together with the corresponding evidences based on the results of SignalP, TMHMM, GPI SOM and WoLF PSORT analyses, and details of functional annotation based on BLASTX similarity search. Click here for file

Additional file 12 **Multiple sequence alignment of EtSAGs**. Alignment of amino acid sequences encoded by EtSAG sequences (a) A family and (b) B family. (-) represents a gap, (*) represents the same residue, (:) represents conserved residue, and (.) represents partially conserved residue. Conserved cysteine residues are coloured in grey. Click here for file

Additional file 13 **Summary of bioinformatic tools used in this study**. List of bioinformatic tools used in this study together with their usage and references. Click here for file

## References

[B1] Shirley MW Smith AL Tomley FM The biology of avian *Eimeria *with an emphasis on their control by vaccination Adv Parasitol 2005 60 285 330 16230106 10.1016/S0065-308X(05)60005-X

[B2] Chapman HD Shirley MW The Houghton strain of *Eimeria tenella*: A review of the type strain selected for genome sequencing Avian Pathol 2003 32 115 127 10.1080/0307945021000071588 12745365

[B3] McDonald V Shirley MW The endogenous development of virulent strains and attenuated precocious lines of *Eimeria tenella *and *E. necatrix* J Parasitol 1987 73 993 997 10.2307/3282523 3656015

[B4] Rose M Hesketh P Immunity to coccidiosis: stages of the life-cycle of *Eimeria maxima *which induce, and are affected by, the response of the host Parasitol 1976 73 25 37 10.1017/S0031182000051295 967527

[B5] Shirley MW Eckert J, Braun R, Shirley MW, Coudert P *Eimeria *species and strains of chickens Guidelines on techniques in coccidiosis research 1995 Luxemborg, European Commission 1 24

[B6] Wan KL Chong SP Ng ST Shirley MW Tomley FM Jangi MS A survey of genes in *Eimeria tenella *merozoites by EST sequencing Int J Parasitol 1999 29 1885 1892 10.1016/S0020-7519(99)00160-5 10961844

[B7] Ng ST Jangi MS Shirley MW Tomley FM Wan KL Comparative EST analyses provide insights into gene expression in two asexual developmental stages of *Eimeria tenella* Exp Parasitol 2002 101 168 173 10.1016/S0014-4894(02)00109-1 12427472

[B8] Li L Brunk BP Kissinger JC Pape D Tang K Cole RH Martin J Wylie T Dante M Fogarty SJ Howe DK Liberator P Diaz C Anderson J White M Jerome ME Johnson EA Radke JA Stoeckert CJ Jr Waterston RH Clifton SW Roos DS Sibley LD Gene discovery in the apicomplexa as revealed by EST sequencing and assembly of a comparative gene database Genome Res 2003 13 443 54 10.1101/gr.693203 12618375 PMC430278

[B9] Aoki K Yano K Suzuki A Kawamura S Sakurai N Suda K Kurabayashi A Suzuki T Tsugane T Watanabe M Ooga K Torii M Narita T Shin-I T Kohara Y Yamamoto N Takahashi H Watanabe Y Egusa M Kodama M Ichinose Y Kikuchi M Fukushima S Okabe A Arie T Sato Y Yazawa K Satoh S Omura T Ezura H Shibata D Large-scale analysis of full-length cDNAs from the tomato (*Solanum lycopersicum*) cultivar Micro-TOM, a reference system for the Solanaceae genomics BMC Genomics 2010 11 210 10.1186/1471-2164-11-210 20350329 PMC2859864

[B10] Lal K Bromley E Oakes R Prieto JH Sanderson SJ Kurian D Hunt L Yates JR Wastling JM Sinden RE Tomley FM Proteomic comparison of four *Eimeria tenella *life-cycle stages: unsporulated oocyst, sporulated oocyst, sporozoite and second-generation merozoite Proteomics 2009 9 4566 4576 10.1002/pmic.200900305 19795439 PMC2947549

[B11] Wakaguri Y Suzuki Y Sasaki M Sugano S Watanabe J Inconsistencies of genome annotations in apicomplexan parasites revealed by 5'-end-one-pass and full-length sequences of oligo-capped cDNAs BMC Genomics 2009 10 312 10.1186/1471-2164-10-312 19602295 PMC2722674

[B12] Yamagishi J Wakaguri H Sugano S Kawano S Fujisaki K Sugimoto C Watanabe J Suzuki Y Kimata I Xuan X Construction and analysis of full-length cDNA library of *Cryptosporidium parvum* Parasitol Int 2011 60 199 202 10.1016/j.parint.2011.03.001 21397714

[B13] Pickering BM Willis AE The implications of structured 5' untranslated regions on translation and disease Semin Cell Dev Biol 2005 16 39 47 10.1016/j.semcdb.2004.11.006 15659338

[B14] Grillo G Turi A Licciulli F Mignone F Liuni S Banfi S Gennarino VA Horner DS Pavesi G Picardi E Pesole G UTRdb and UTRsite (RELEASE 2010): a collection of sequences and regulatory motifs of the untranslated regions of eukaryotic mRNAs Nucl Acids Res 2009 38 D75 D80 19880380 10.1093/nar/gkp902 PMC2808995

[B15] *Eimeria tenella *on GeneDB http://www.genedb.org/Homepage/Etenella

[B16] Ling KH Rajandream MA Rivailler P Ivens A Yap SJ Madeira AMBN Mungall K Billington K Yee WY Bankier AT Carroll F Durham AM Peters N Loo SS Mat-Isa MN Novaes J Quail M Rosli R Shamsudin MN Sobreira TJP Tivey AR Wai SF White S Wu X Kerhornou A Blake D Mohamed R Shirley M Gruber A Berriman M Tomley F Dear PH Wan KL Sequencing and analysis of chromosome 1 of *Eimeria tenella *reveals a unique segmental organization Genome Res 2007 17 311 319 10.1101/gr.5823007 17284678 PMC1800922

[B17] Loo SS Blake DP Mohd-Adnan A Mohamed R Wan KL *Eimeria tenella *glucose-6-phosphate isomerase: molecular characterization and assessment as a target for anti-coccidial control Parasitol 2010 137 1169 1177 10.1017/S0031182010000119 20233491

[B18] Gur-Arie R Cohen CJ Eitan Y Shelef L Hallerman EL Kashi Y Simple sequence repeats in *Escherichia coli*: abundance, distribution, composition, and polymorphism Genome Res 2000 10 62 71 10645951 PMC310497

[B19] Toth G Gaspari Z Jurka J Microsatellites in different eukaryotic genomes: survey and analyis Genome Res 2000 10 967 981 10.1101/gr.10.7.967 10899146 PMC310925

[B20] Morgante M Olivieri AM PCR-amplified microsatellites as markers in plant genetics Plant J 1993 3 175 182 10.1111/j.1365-313X.1993.tb00020.x 8401603

[B21] Powell W Morgante M Andre C Henfey M Vogel J Tingy S Rafalsky A The comparison of RFLP, RAPD, AFLP and SSR (microsatellite) markers for germplasm analysis Mol Breed 1996 2 225 238 10.1007/BF00564200

[B22] McCouch SR Teytelman L Xu YB Lobos KB Clare K Walton M Fu BY Maghirang R Li ZK Xing YZ Zhang QF Kono I Yano M Fjellstrom R DeClerck G Schneider D Cartinhour S Ware D Stein L Development and mapping of 2240 new SSR markers for rice (*Oryza sativa *L.) DNA Res 2002 9 199 207 10.1093/dnares/9.6.199 12597276

[B23] Somers DJ Isaac P Edwards K A high-density microsatellites consensus map for bread wheat (*Triticum aestivum *L.) Theor Appl Genet 2004 109 1105 1114 10.1007/s00122-004-1740-7 15490101

[B24] Innan H Terauchi R Miyashita NT Microsatellite polymorphism in natural populations of the wild plant *Arabidopsis thaliana* Genetic 1997 146 1441 1452 10.1093/genetics/146.4.1441 PMC1208087 9258686

[B25] Garza JC Slatkin M Freimer NB Microsatellite allele frequencies in humans and chimpanzees, with implications for constraints on allele size Mol Biol Evol 1995 12 594 603 7659015 10.1093/oxfordjournals.molbev.a040239

[B26] MacHugh DE Shriver MD Loftus RT Cunningham P Bradley DG Microsatellite DNA variation and the evolution, domestication and phylogeography of taurine and Zebu cattle (*Bos taurus *and *Bos indicus*) Genetics 1997 146 1071 1086 9215909 10.1093/genetics/146.3.1071 PMC1208036

[B27] Kashi Y King D Soller M Simple sequence repeats as a source of quantitative genetic variation Trends Genet 1997 13 74 78 10.1016/S0168-9525(97)01008-1 9055609

[B28] Santi L Wang Y Stile MR Berendzen K Wanke D Roig C Pozzi C Muller K Muller J Rohde W Salamini F The GA octodinucleotide repeat binding factor BBR participates in the transcriptional regulation of the homeobox gene Bkn3 Plant J 2003 34 813 816 10.1046/j.1365-313X.2003.01767.x 12795701

[B29] Saveliev A Everett C Sharpe T Webster Z Frestenstein R DNA triplet repeats mediate heterochromatin-protein-1-sensitive variegated gene silencing Nature 2003 422 909 913 10.1038/nature01596 12712207

[B30] Chiapello H Ollivier E Landes-Devauchelle C Nitschke P Risler JL Codon usage as a tool to predict the cellular location of eukaryotic ribosomal proteins and aminoacyl-tRNA synthetases Nucl Acids Res 1999 27 2848 2851 10.1093/nar/27.14.2848 10390524 PMC148497

[B31] Fickett JW Recognition of protein coding regions in DNA sequences Nucl Acids Res 1982 10 5303 5318 10.1093/nar/10.17.5303 7145702 PMC320873

[B32] Osawa S Jukes TH Watanabe K Muto A Recent evidence for evolution of the genetic code Microbiol Rev 1992 56 229 264 1579111 10.1128/mr.56.1.229-264.1992 PMC372862

[B33] Phillips GJ Arnold J Ivarie R The effect of codon usage on the oligonucleotide composition of the *E. coli *genome and identification of over and under presented sequences by Markov chain analysis Nucl Acids Res 1987 15 2627 2638 10.1093/nar/15.6.2627 3550700 PMC340673

[B34] Zhang S Zubay G Goldman E Low-usage codons in *Escherichia coli*, yeast, fruit fly and primates Gene 1991 105 61 72 10.1016/0378-1119(91)90514-C 1937008

[B35] Ellis J Griffin H Morrison D Johnson AM Analysis of dinucleotide frequency and codon usage in the phylum Apicomplexa Gene 1993 126 163 170 10.1016/0378-1119(93)90363-8 8482530

[B36] Seeber F Consensus sequence of translational initiation sites from *Toxoplasma gondii* Parasitol Res 1997 83 309 311 10.1007/s004360050254 9089733

[B37] Kozak M The scanning model for translation: an update J Cell Biol 1989 108 229 241 10.1083/jcb.108.2.229 2645293 PMC2115416

[B38] Vercammen M Scorza T Huygen K De Braekeleer J Diet R Jacobs D Saman E Verschueren H DNA vaccination with genes encoding *Toxoplasma gondii *antigens GRA1, GRA7, and ROP2 induces partially protective immunity against lethal challenge in mice Infect Immun 2000 68 1 38 45 10.1128/IAI.68.1.38-45.2000 10603366 PMC97099

[B39] Moorthy VS Good MF Hill AV Malaria vaccine developments Lancet 2004 363 9403 150 156 10.1016/S0140-6736(03)15267-1 14726170

[B40] Jenkins MC Advances and prospects for subunit vaccines against protozoa of veterinary importance Vet Parasitol 2001 101 3-4 291 310 10.1016/S0304-4017(01)00557-X 11707303

[B41] Klotz C Gehre F Lucius R Pogonka T Identification of *Eimeria tenella *genes encoding for secretory proteins and evaluation of candidates by DNA immunisation studies in chickens Vaccine 2007 25 36 6625 6634 10.1016/j.vaccine.2007.06.048 17675183

[B42] Ropert C Gazzinelli RT Signalling of immune system cells by glycosylphosphatidylinositol (GPI) anchor and related structures derived from parasitic protozoa Curr Opin Microbiol 2000 3 395 403 10.1016/S1369-5274(00)00111-9 10972501

[B43] Chow YP Wan KL Blake DP Tomley F Nathan S Immunogenic *Eimeria tenella *glycosylphosphatidylinositol-anchored surface antigens (SAGs) induce inflammatory responses in avian macrophages PLoS ONE 2011 6 9 e25233 10.1371/journal.pone.0025233 21980402 PMC3182191

[B44] Tabares E Ferguson D Clark J Soon PE Wan KL Tomley FM *Eimeria tenella *sporozoites and merozoites diffrentially express glycosylphosphatidylinositol-anchored variant surface proteins Mol Biochem Parasitol 2004 135 123 132 10.1016/j.molbiopara.2004.01.013 15287593

[B45] Klotz C Marhöfer RJ Selzer PM Lucius R Pogonka T *Eimeria tenella*: identification of secretory and surface proteins from expressed sequence tags Exp Parasitol 2005 111 14 23 10.1016/j.exppara.2005.04.005 15936018

[B46] Shirley MW Ivens A Gruber A Madeira AMBN Wan KL Dear PH Tomley FM The *Eimeria *genome projects: A sequence of events Trends Parasitol 2004 20 199 201 10.1016/j.pt.2004.02.005 15105014

[B47] Maruyama K Sugano S Oligo-capping: a simple method to replace the cap structure of eukaryotic mRNAs with oligoribonucleotides Gene 1994 138 171 174 10.1016/0378-1119(94)90802-8 8125298

[B48] Ewing B Green P Base-calling of automated sequencer traces using phred. II. Error probabilities Genome Res 1998 8 186 194 9521922

[B49] Ewing B Hillier L Wendl M Green P Base-calling of automated sequencer traces using phred. I. Accuracy assessment Genome Res 1998 8 175 185 9521921 10.1101/gr.8.3.175

[B50] Burke J Davison D Hide WA D2_cluster: a validated method for clustering EST and full-length cDNA sequences Genome Res 1999 9 1135 1142 10.1101/gr.9.11.1135 10568753 PMC310833

[B51] Miller RT Christoffels AG Gopalakrishnan C Burke J Ptitsyn AA Broveak TR Hide WA A comprehensive approach to clustering of expressed human gene sequence: The Sequence Tag Alignment and Consensus Knowledgebase Genome Res 1999 9 1143 1155 10.1101/gr.9.11.1143 10568754 PMC310831

[B52] Rozen S Skaletsky HJ Krawetz S, Misener S Primer3 on the WWW for general users and for biologist programmers Bioinformatics methods and protocols: Methods in molecular biology 2000 Humana Press, Totowa, NJ 365 386 10.1385/1-59259-192-2:365 10547847

[B53] Altschul J Madden TL Schaffer AA Zhang J Zhang Z Miller W Lipman DJ Gapped BLAST and PSI-BLAST: a new generation of protein database search programs Nucl Acids Res 1997 25 3389 3402 10.1093/nar/25.17.3389 9254694 PMC146917

[B54] Conesa A Götz S García-Gόmez JM Terol J Talόn M Robles M Blast2GO: a universal tool for annotation, visualization and analysis in functional genomics research Bioinformatics 2005 21 3674 3676 10.1093/bioinformatics/bti610 16081474

[B55] ssahaEST http://www.sanger.ac.uk/resources/software/ssahaest/

[B56] Rutherford K Parkhill J Crook J Horsnell T Rice P Rajandream MA Barrell B Artemis: sequence visualization and annotation Bioinformatics 2000 16 944 945 10.1093/bioinformatics/16.10.944 11120685

[B57] ORF Finder http://www.ncbi.nlm.nih.gov/gorf/

[B58] MISA http://pgrc.ipk-gatersleben.de/misa/

[B59] CodonW http://codonw.sourceforge.net/

[B60] Crooks GE Hon G Cahndonia JM Brenner S WebLogo: A sequence logo generator Genome Res 2004 14 1188 1190 10.1101/gr.849004 15173120 PMC419797

[B61] Petersen TN Brunak S von Heijne G Nielsen H SignalP 4.0: discriminating signal peptides from transmembrane regions Nat Methods 2011 8 785 786 10.1038/nmeth.1701 21959131

[B62] Krogh A Larsson B von Heijne G Sonnhammer ELL Predicting transmembrane protein topology with a hidden Markov model: application to complete genomes J Mol Biol 2001 305 567 580 10.1006/jmbi.2000.4315 11152613

[B63] Frankhauser N Maser P Identification of GPI anchor attachment signals by a Kohonen self-organizing map Bioinformatics 2005 21 1846 1852 10.1093/bioinformatics/bti299 15691858

[B64] Horton P Park KJ Obayashi T Fujita N Harada H Adams-Collier CJ Nakai K WoLF PSORT: protein localization predictor Nucl Acids Res 2007 35 W585 W587 10.1093/nar/gkm259 17517783 PMC1933216

