# Supplementary material for: Characterisation of full-length cDNA sequences provides insights into the Eimeria tenellatranscriptome
Source: BMC Genomics. 2012 Jan 13;13:21. doi: 10.1186/1471-2164-13-21 (PMC3315734; doi:10.1186/1471-2164-13-21)
Supplement: Additional file 4 — Mapping of Eimeria tenella transcripts to chromosome 1 sequence. Number of Eimeria tenella unique and full-length transcripts mapped to the predicted coding, P- and R-regions of chromosome 1. [file 1471-2164-13-21-S4.DOCX]

**Additional file 4. Mapping of *Eimeria* *tenella* transcripts to chromosome 1 sequence**

|  | Unique  transcripts | Full-length  transcripts |
| --- | --- | --- |
| Total transcript mapped | 13 | 2 |
| Transcripts mapped to predicted coding regions | 13 | 2 |
| Consistent with predicted coding regions* | 4 | 1 |
| Inconsistent with predicted regions | 9 | 1 |
| Transcripts mapped to P-region | 7 | 1 |
| Transcripts mapped to R-region | 6 | 1 |

*With two or less exons being different
