# Supplementary figures and images for: Characterisation of full-length cDNA sequences provides insights into the Eimeria tenellatranscriptome
Source: BMC Genomics. 2012 Jan 13;13:21. doi: 10.1186/1471-2164-13-21 (PMC3315734; doi:10.1186/1471-2164-13-21)

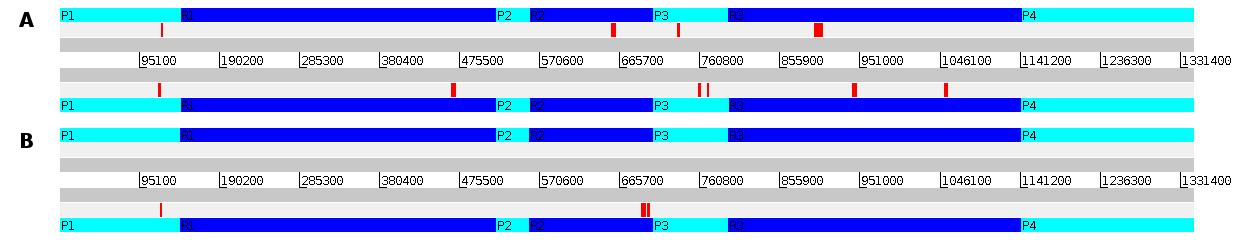

Supplement: Additional file 5 — Position of Eimeria tenella transcripts on chromosome 1 sequence. Graphical representation of the positions of Eimeria tenella (A) unique and (B) full-length transcripts that mapped to chromosome 1. Segmentation of the chromosome is shown in cyan (P-region) and blue (R-region). Positions of transcripts are represented by red vertical lines. [file 1471-2164-13-21-S5.PNG]

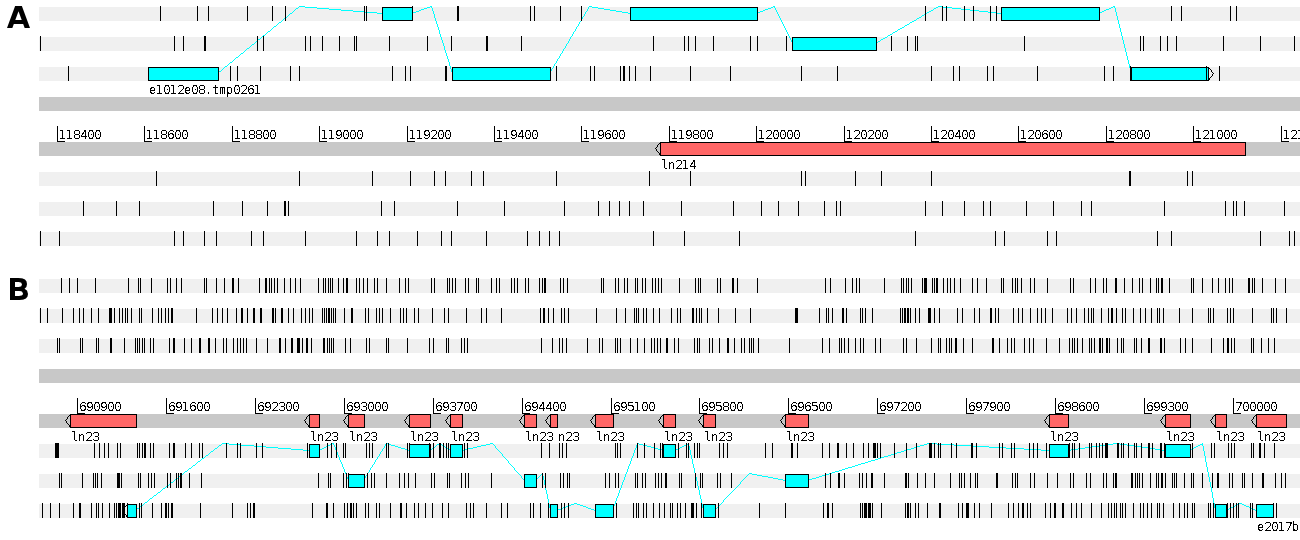

Supplement: Additional file 6 — Alignment of Eimeria tenella full-length transcripts to chromosome 1. Graphical representation of the alignment of full-length transcripts (A) ln214_Etm109D12 and (B) ln23_Etm023C06 to the respective predicted genes on chromosome 1. Mapped full-length transcripts are shown in red while predicted genes are shown in cyan. [file 1471-2164-13-21-S6.PNG]

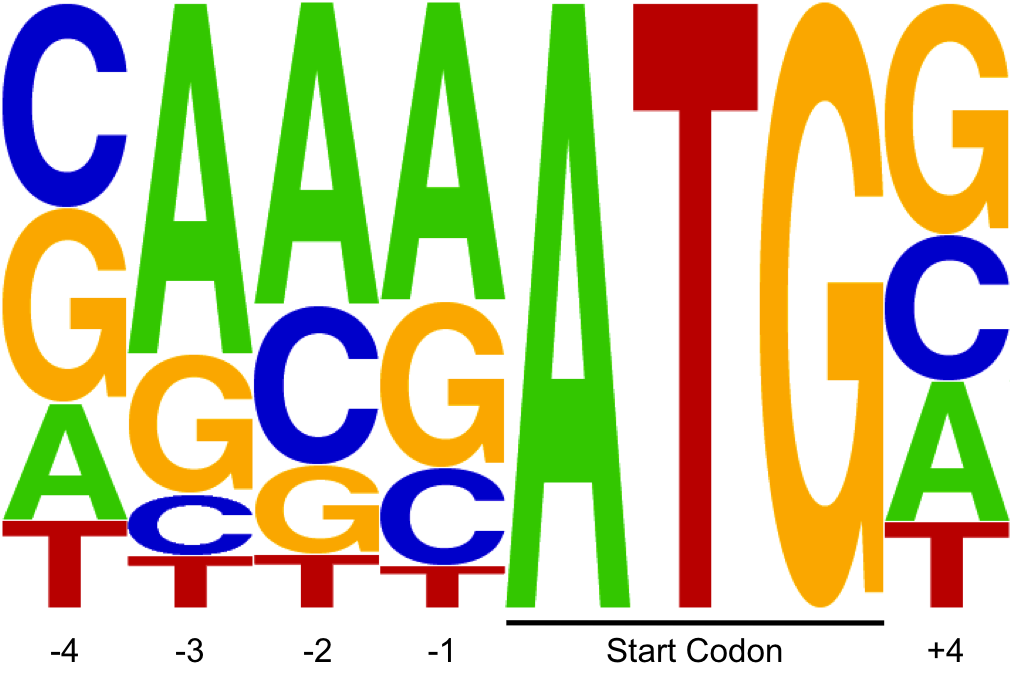

Supplement: Additional file 9 — Kozak motif profile from full-length cDNA sequences of Eimeria tenella. Graphical representation of the consensus sequence of translational initiation sites (the Kozak sequence) based on the alignment of start codons derived from Eimeria tenella full-length cDNA sequences. [file 1471-2164-13-21-S9.PNG]
