# Supplementary material for: Characterisation of full-length cDNA sequences provides insights into the Eimeria tenellatranscriptome
Source: BMC Genomics. 2012 Jan 13;13:21. doi: 10.1186/1471-2164-13-21 (PMC3315734; doi:10.1186/1471-2164-13-21)
Supplement: Additional file 7 — Details of SSR motif distribution in full-length cDNA sequences of Eimeria tenella. List of SSR motifs identified in Eimeria tenella full-length cDNA sequences together with their repeat number, copy number and total length within the 3'UTR, ORF and 5'UTR. [file 1471-2164-13-21-S7.DOCX]

| **Additional file 7. Details of SSR motif distribution in full-length cDNA sequences of *Eimeria tenella*** | | | | | | | | | |
| --- | --- | --- | --- | --- | --- | --- | --- | --- | --- |
| SSR type | 5'UTR | | | ORF | | | 3'UTR | | |
|  | Repeat number | Copy number | Total SSR length (bp) | Repeat number | Copy number | Total SSR length (bp) | Repeat number | Copy number | Total SSR length (bp) |
| A | 7 | 84 | 84 | 1 | 10 | 10 | 5 | 87 | 87 |
| G | 0 | 0 | 0 | 3 | 34 | 34 | 0 | 0 | 0 |
| T | 1 | 11 | 11 | 0 | 0 | 0 | 1 | 10 | 10 |
| AT | 0 | 0 | 0 | 0 | 0 | 0 | 6 | 47 | 94 |
| TC | 0 | 0 | 0 | 0 | 0 | 0 | 1 | 6 | 12 |
| GA | 0 | 0 | 0 | 1 | 6 | 12 | 0 | 0 | 0 |
| AAT | 0 | 0 | 0 | 0 | 0 | 0 | 2 | 10 | 30 |
| AGA | 0 | 0 | 0 | 3 | 24 | 72 | 0 | 0 | 0 |
| CAA | 0 | 0 | 0 | 2 | 10 | 30 | 0 | 0 | 0 |
| CAC | 0 | 0 | 0 | 2 | 12 | 36 | 0 | 0 | 0 |
| CAG | 95 | 685 | 2055 | 149 | 987 | 2961 | 81 | 541 | 1623 |
| CCG | 2 | 15 | 45 | 0 | 0 | 0 | 0 | 0 | 0 |
| CCT | 0 | 0 | 0 | 1 | 8 | 24 | 0 | 0 | 0 |
| CGG | 0 | 0 | 0 | 2 | 12 | 36 | 24 | 152 | 456 |
| CTG | 36 | 249 | 747 | 26 | 156 | 468 | 27 | 183 | 549 |
| GAG | 0 | 0 | 0 | 2 | 12 | 36 | 0 | 0 | 0 |
| GAT | 0 | 0 | 0 | 1 | 9 | 27 | 0 | 0 | 0 |
| AAAT | 0 | 0 | 0 | 0 | 0 | 0 | 1 | 5 | 20 |
| AGCT | 0 | 0 | 0 | 0 | 0 | 0 | 15 | 95 | 380 |
| GCAT | 0 | 0 | 0 | 0 | 0 | 0 | 1 | 5 | 20 |
| GGCT | 0 | 0 | 0 | 0 | 0 | 0 | 1 | 6 | 24 |
| TTAT | 0 | 0 | 0 | 0 | 0 | 0 | 3 | 17 | 68 |
| AACCCT | 0 | 0 | 0 | 1 | 9 | 54 | 0 | 0 | 0 |
| AAGTCG | 1 | 5 | 30 | 0 | 0 | 0 | 0 | 0 | 0 |
| CCACAG | 0 | 0 | 0 | 2 | 13 | 78 | 0 | 0 | 0 |
| GACGGC | 0 | 0 | 0 | 1 | 15 | 90 | 0 | 0 | 0 |
| GAGCCA | 0 | 0 | 0 | 1 | 11 | 66 | 0 | 0 | 0 |
| TCGGCT | 0 | 0 | 0 | 1 | 9 | 54 | 0 | 0 | 0 |
| TCTACA | 0 | 0 | 0 | 1 | 6 | 36 | 0 | 0 | 0 |
| AAACCCT | 2 | 10 | 70 | 0 | 0 | 0 | 0 | 0 | 0 |
| GATAAGGAG | 0 | 0 | 0 | 1 | 6 | 54 | 0 | 0 | 0 |
| GGAAAAGAA | 0 | 0 | 0 | 0 | 0 | 0 | 1 | 6 | 54 |
| CAGCTAGCCA | 0 | 0 | 0 | 0 | 0 | 0 | 1 | 5 | 50 |
| Total | 144 | 1059 | 3042 | 201 | 1349 | 4178 | 170 | 1175 | 3477 |
