# Supplementary material for: Characterisation of full-length cDNA sequences provides insights into the Eimeria tenellatranscriptome
Source: BMC Genomics. 2012 Jan 13;13:21. doi: 10.1186/1471-2164-13-21 (PMC3315734; doi:10.1186/1471-2164-13-21)
Supplement: Additional file 8 — Comparison of SSR motifs in Eimeria tenella, Toxoplasma gondii and Cryptosporidium parvum full-length cDNA sequences. List of SSR motifs identified in Eimeria tenella, Toxoplasma gondii and Cryptosporidium parvum full-length cDNA sequences together with their repeat number, copy number, total length and the percentage of their total length over the total length of the respective full-length cDNA sequences. [file 1471-2164-13-21-S8.DOCX]

**Additional file 8. Comparison of SSR motifs in *Eimeria tenella, Toxoplasma gondii* and *Cryptosporidium parvum* full-length cDNA sequences**

| SSR type | *E. tenella* | | | | *C. parvum* | | | | *T. gondii* | | | |
| --- | --- | --- | --- | --- | --- | --- | --- | --- | --- | --- | --- | --- |
|  | Repeat number | Copy number | Total SSR length (bp) | Percentage of total sequence length^a^ (%) | Repeat number | Copy number | Total SSR length (bp) | Percentage of total sequence length^b^ (%) | Repeat number | Copy number | Total SSR length (bp) | Percentage of total sequence length^c^ (%) |
| Mononucleotide | 18 | 236 | 236 | 0.03 | 137 | 1603 | 1603 | 0.18 | 15 | 163 | 163 | 0.01 |
| Dinucleotide | 8 | 59 | 118 | 0.02 | 10 | 64 | 128 | 0.01 | 18 | 137 | 274 | 0.02 |
| Trinucleotide | 455 | 3066 | 9198 | 1.29 | 37 | 239 | 717 | 0.08 | 8 | 40 | 120 | 0.01 |
| Tetranucleotide | 21 | 128 | 512 | 0.07 | 1 | 5 | 20 | 0.00 | 0 | 0 | 0 | 0 |
| Hexanucleotide | 8 | 68 | 408 | 0.06 | 4 | 24 | 144 | 0.02 | 0 | 0 | 0 | 0 |
| Heptanucleotide | 2 | 10 | 70 | 0.00 | 0 | 0 | 0 | 0 | 0 | 0 | 0 | 0 |
| Nonanucleotide | 2 | 12 | 108 | 0.02 | 0 | 0 | 0 | 0 | 0 | 0 | 0 | 0 |
| Decanucleotide | 1 | 5 | 50 | 0.01 | 0 | 0 | 0 | 0 | 0 | 0 | 0 | 0 |
| Total | 515 | 3584 | 10700 | 1.50 | 189 | 1935 | 2612 | 0.29 | 41 | 340 | 557 | 0.04 |

^a^Calculated based on the total length of 433 full-length cDNAs (713,152 bp)

^b^Calculated based on the total length of 644 full-length cDNAs (900,852 bp)

^c^Calculated based on the total length of 732 full-length cDNAs (1,126,449 bp)
