# Supplementary material for: Characterisation of full-length cDNA sequences provides insights into the Eimeria tenellatranscriptome
Source: BMC Genomics. 2012 Jan 13;13:21. doi: 10.1186/1471-2164-13-21 (PMC3315734; doi:10.1186/1471-2164-13-21)
Supplement: Additional file 12 — Multiple sequence alignment of EtSAGs. Alignment of amino acid sequences encoded by EtSAG sequences (a) A family and (b) B family. (-) represents a gap, (*) represents the same residue, (:) represents conserved residue, and (.) represents partially conserved residue. Conserved cysteine residues are coloured in grey. [file 1471-2164-13-21-S12.DOCX]

(a)

EtSAG4 -----------MARVAFFSLVFVPLVFNQAIAQQQAATPDAKKLN----C 35

EtSAG6 ------MLPLRIPSVFSASIFLLSVSYLGTSQQSAPTIKYTASLGGGAKC 44

cn425_Etm041A07 MDQSTEMFRMNLATFLSVSLLWLSEKSSQ--AAAATTVKYTAKLGSSVEC 48

. .:. :. : . . *

EtSAG4 LEAMNALRTAAGLAEFKEASTATQILPEKAVEKDATVKPGTLWEEICPKV 85

EtSAG6 LSEVNAARGAAGLKNFAEATN-DKKLSAP-SDDLENDT---EWKKVCEHL 89

cn425_Etm041A07 LGEVNNARQAAGLANFIKATNDGDKISDPGSADLTDG----DWKEICEYL 94

: :* * **** * *: :. *..:* :

EtSAG4 RGTEPDNITEAKKLT----GTFAYYPVADGKKDCNAAVEYWKGGFSLFKN 131

EtSAG6 IPTQKEPVEATSGTNPFEKGTYAFKSLTTAEPNCKEIVNYWKAAFKNFT- 138

cn425_Etm041A07 VPTQPEAHSSQAATETFKDGTYAFKALTAEQPNCTETIDYWKAAYKNFT- 143

: * :*: : .* ::*** .: *.

EtSAG4 EIPPEFTEANKTTVYNDRAVSFVALYNPKPDPVVSCVLLQCPTATSPGVP 181

EtSAG6 GLPPSESQAG-DLYKSYNNVSFVALYNTSSNATADCQVVTCTKTTTPGDS 187

cn425_Etm041A07 GLPPSKKGTG-KLYENQDNVSFVALYNASSNATADCRVVTCTQKTSAAAV 192

:** . . .*******. ... ..* .: *.

EtSAG4 GAG---RRLS------SSSTTVEAVICLTNPAALT-ENAAPFKEDEWKKI 221

EtSAG6 SIRDSPSGSQE---------YGYAMICKTMPAAFADKNSAPFTQDQWDRI 228

cn425_Etm041A07 SASVSSGDSGD------TTKLGYALICKTMPTAFGNGSIAPFTQEQWDKI 236

.::* * *:.: **.:: * :*

EtSAG4 VEAISGNKSE--VSPVGPSVALVSATAIAVFALF 253

EtSAG6 ISSLTGSASAAIPGFGAFFIVVLSMAVL------ 256

cn425_Etm041A07 KYSLTGSASIAVPSLVALVIVAFGITAL------ 264

:: *. . . : :

(b)

cn170_Etm087H03 MLRPGLLACYIGLLAGAATASFSGAIITRSANTNPHYDIVDAETAFVQNATTP----IVA 56

cn1314_Etm004E01 MTYVGLLACYAGLLASAAAPHFSSAISLRAGTATSQKSSLRTNLFASGQDLLRTTTAPTA 60

cn172_Etm002E02 MRRPGLLTCYISLLAGAATAHFSAAVITRSANTNPHYGIVETEAAFLQAAATP----IET 56

cn840_Etm111G02 MTHLGLLACYAGLLASAAAPHFSLALSLRSGTAASQQSSLSANLFASGQVSLR--AAPTG 58

ln503_Etm033E09 MPQFGLFTCYAGLLAGVAASSFSGAVPIRFVTASPHQRSLDTKLSSFAQESNP----PTA 56

ln578_Etm091E10 MTHIGLLACFGGLLASATAPHITLALSLRSGTAASQQSSLRTNLFASGQDLQGTTTAPTG 60

* **::*: .** ..::. :: *: : : .: : :

cn170_Etm087H03 EDATTACLPTMNMLRVLNLRDQALDALQPETGG--ASEDEEREEQGEHTKSKTTAEIAKE 114

cn1314_Etm004E01 NEKTQDCLEIINTLRKENLQDLLGTLTKAEESD---VTASLKKIKIEGSDELSTAKIAAK 117

cn172_Etm002E02 TDATTACLPTMNMLRVLNLRDQALEALQPETGGTTVQEGEEEDDQEELTKSNTVAEIAKG 116

cn840_Etm111G02 NEITADCLDTINKLRNENIKDLLGTLTKAEDSD---VTASLKTIPVADAASLTTATIAAK 115

ln503_Etm033E09 EDKTDACLPILNGLRTEGLSAVLVELKKAEEQD---VSGSLTGLLPE--SKTKVTDIAAE 111

ln578_Etm091E10 ADKTEECLDIINKLRDENLKDLLGTLTKAKEND---VTASLKEIGIEDPAEPTTAKIAEK 117

: * ** :* ** .: :.. . .. **

cn170_Etm087H03 LAGTKAETCEKGATADAKTHTGLVIPFEYSTVFDCGSLIQGHFAAGLSHMQESNFDPATG 174

cn1314_Etm004E01 LAGSDAQNCESGESANAKTYPGLVIPFPHTTDFDCNTLIQATYTAGLDHLKQSNFEPSTG 177

cn172_Etm002E02 LAGTDADKCEAGATANAKTHTGLVIPFEYSTTFDCGSLIQDHFTTGLTHIQESNFDPATG 176

cn840_Etm111G02 LAGDSVDTCASGGNADAKTYPGLVIPFTHDKDFDCDALIQATYTAGLNQLKQSNFEPSKG 175

ln503_Etm033E09 LAGSDKASCDGAAIKDSK-YPGLVIPFDHSTEFDCEALINDSFYAGLSHLEENNYDPSAE 170

ln578_Etm091E10 LAGNDVQSCESGKSANAKTYPGLVIPFAHDRDFDCKALIQATYTAGLDHLKQLNFEPSTG 177

*** . .* . ::* :.****** : *** :**: : :** :::: *::*:

cn170_Etm087H03 AYDTGKAPFDNLSASNVANIMWSKSTKASCAVTKNCQAGHNVLYCRLVEPITSQ-DKPFT 233

cn1314_Etm004E01 TYDVDKTPINNVDASNVAFLLSAKSTKVSCAATEDCAGGHDVLFCYFIDPLQSG-DQAFT 236

cn172_Etm002E02 TYDTGKAPFDNLSASNVANIMWSKSKKASCAVTNNCRAGHNVLYCRFVDAIKPE-DKPFT 235

cn840_Etm111G02 TYDATKAPFDNVDASNVAFLLSAKSKKVSCAATKNCNAGHDVLFCYFIEPLRNG-DQPFT 234

ln503_Etm033E09 SSQLGVAPLDNRAAQNLAAIVYDESEKVACAATTDCAAGNNVLFCYFVNPLKKETKKPIN 230

ln578_Etm091E10 TYNAEKAPFDNIDASNVAFLLSAKSKTVSCAATENCEGGHDVLFCYFMEPLQKG-QKPFT 236

: :*::* *.*:* :: :* ..:**.* :* .*::**:* :::.: . .:.

cn170_Etm087H03 TELYEALLQRQAGSSSIALTSIATTFFCAAWLLST 268

cn1314_Etm004E01 TELYNALWGLEAGAASISVPSVATILLVLALGIWN 271

cn172_Etm002E02 TELYEALLQRQAGSASIALTSIATTFFCAALLLLS 270

cn840_Etm111G02 TELYNALWGLEAGAASTAVPSVATVLLTLALIIQP 269

ln503_Etm033E09 AEVYEVLLKRPRGSASITIPAIAATLLSLALAMLS 265

ln578_Etm091E10 TELYNALWGLETGTASISVPNAFTALSVLALMIRT 271

:*:*:.* *::* :.. : : * :
