# Supplementary material for: Characterisation of full-length cDNA sequences provides insights into the Eimeria tenellatranscriptome
Source: BMC Genomics. 2012 Jan 13;13:21. doi: 10.1186/1471-2164-13-21 (PMC3315734; doi:10.1186/1471-2164-13-21)
Supplement: Additional file 13 — Summary of bioinformatic tools used in this study. List of bioinformatic tools used in this study together with their usage and references. [file 1471-2164-13-21-S13.DOCX]

**Additional file 13. Summary of bioinformatic tools used in this study**

| **Bioinformatic tool** | **Usage** | **Reference** |
| --- | --- | --- |
| Phred | Quality assessment and filtering | [48,49] |
| StackPACK | Sequence clustering | [50,51] |
| Primer3 | Primer design | [52] |
| BLAST | Sequence similarity search | [53] |
| BLAST2GO | Assignment of GO terms | [54] |
| ssahaEST | Transcript mapping | [55] |
| Artemis | Genome browser | [56] |
| ORF Finder | Prediction of open reading frame | [57] |
| MISA | SSR analysis | [58] |
| CodonW | Codon usage analysis | [59] |
| WebLogo | Generation of sequence logo | [60] |
| SignalP | Prediction of signal peptides | [61] |
| TMHMM | Prediction of transmembrane protein topology | [62] |
| GPI-SOM | Prediction of GPI-anchored proteins | [63] |
| WoLF PSORT | Prediction of protein localisation | [64] |
